# Eight unique basal bodies in the multi-flagellated diplomonad *Giardia lamblia*

**DOI:** 10.1186/s13630-016-0042-4

**Published:** 2016-07-04

**Authors:** Shane G. McInally, Scott C. Dawson

**Affiliations:** Department of Microbiology and Molecular Genetics, University of California Davis, One Shields Avenue, Davis, CA 95616 USA

**Keywords:** *Giardia lamblia*, Basal body, Axonemes, Flagella

## Abstract

*Giardia lamblia* is an intestinal parasitic protist that causes significant acute and chronic diarrheal disease worldwide. *Giardia* belongs to the diplomonads, a group of protists in the supergroup Excavata. Diplomonads are characterized by eight motile flagella organized into four bilaterally symmetric pairs. Each of the eight *Giardia* axonemes has a long cytoplasmic region that extends from the centrally located basal body before exiting the cell body as a membrane-bound flagellum. Each basal body is thus unique in its cytological position and its association with different cytoskeletal features, including the ventral disc, axonemes, and extra-axonemal structures. Inheritance of these unique and complex cytoskeletal elements is maintained through basal body migration, duplication, maturation, and their subsequent association with specific spindle poles during cell division. Due to the complex composition and inheritance of specific basal bodies and their associated structures, *Giardia* may require novel basal body-associated proteins. Thus, protists such as *Giardia* may represent an undiscovered source of novel basal body-associated proteins. The development of new tools that make *Giardia* genetically tractable will enable the composition, structure, and function of the eight basal bodies to be more thoroughly explored.

## Background

*Giardia lamblia* is a single-celled protistan parasite that causes acute and chronic diarrheal disease, primarily in developing countries with inadequate sanitation and water treatment [1, 2]. The life cycle of *Giardia* includes two stages: the proliferative pathogenic trophozoite and the dormant infective cyst. *Giardia* belongs to the diplomonads, a group of protists in the supergroup Excavata whose defining cytological characteristics include eight motile flagella and two nuclei [3]. The discovery of *Giardia* is attributed to Antonie van Leewenhoek, [4] who in 1681 observed teardrop-shaped flagellates in his own stool. More than 300 years later, our understanding of *Giardia* cytoskeletal biology remains rudimentary. This deficit is primarily due to a lack of tools for genetic manipulation; however, improved cytological descriptions and increasing numbers of genomes of *Giardia* species and other related diplomonads are aiding comparisons of the cytoskeletal biology of *Giardia* to other diverse flagellated protists [5].

A recent classification scheme categorized all known eukaryotes into six primary lineages or supergroups: Opisthokonts (e.g., animals, fungi), Amoebozoa, Archaeplastida (e.g., plants and green algae), Rhizaria, Chromalveolata, and the Excavata [6, 7]. Excavate protists have been proposed to be a basal lineage of eukaryotes, closest to the common ancestor of all extant eukaryotes [8, 9]. Yet, the evolutionary diversity within the Excavata represents genetic distances greater than those between plants, animals, and fungi [6]. Molecular phylogenetic support for the monophyly of this group is controversial [10]. All known excavates have flagellated life cycle stages and, as a group, excavates are defined by the presence of posteriorly directed flagella and flagellar root structures associated with the basal bodies [11]. However, excavate biology is quite varied, and diversity within this group encompasses free-living, commensal, and parasitic forms of the following types of protists: Fornicata (diplomonads, oxymonads, and retortamonads), Parabasalia, Euglenozoa (both euglenids and kinetoplastids), Heterolobosea, Jakobida, and Preaxostyla.

**Fig. 1:**
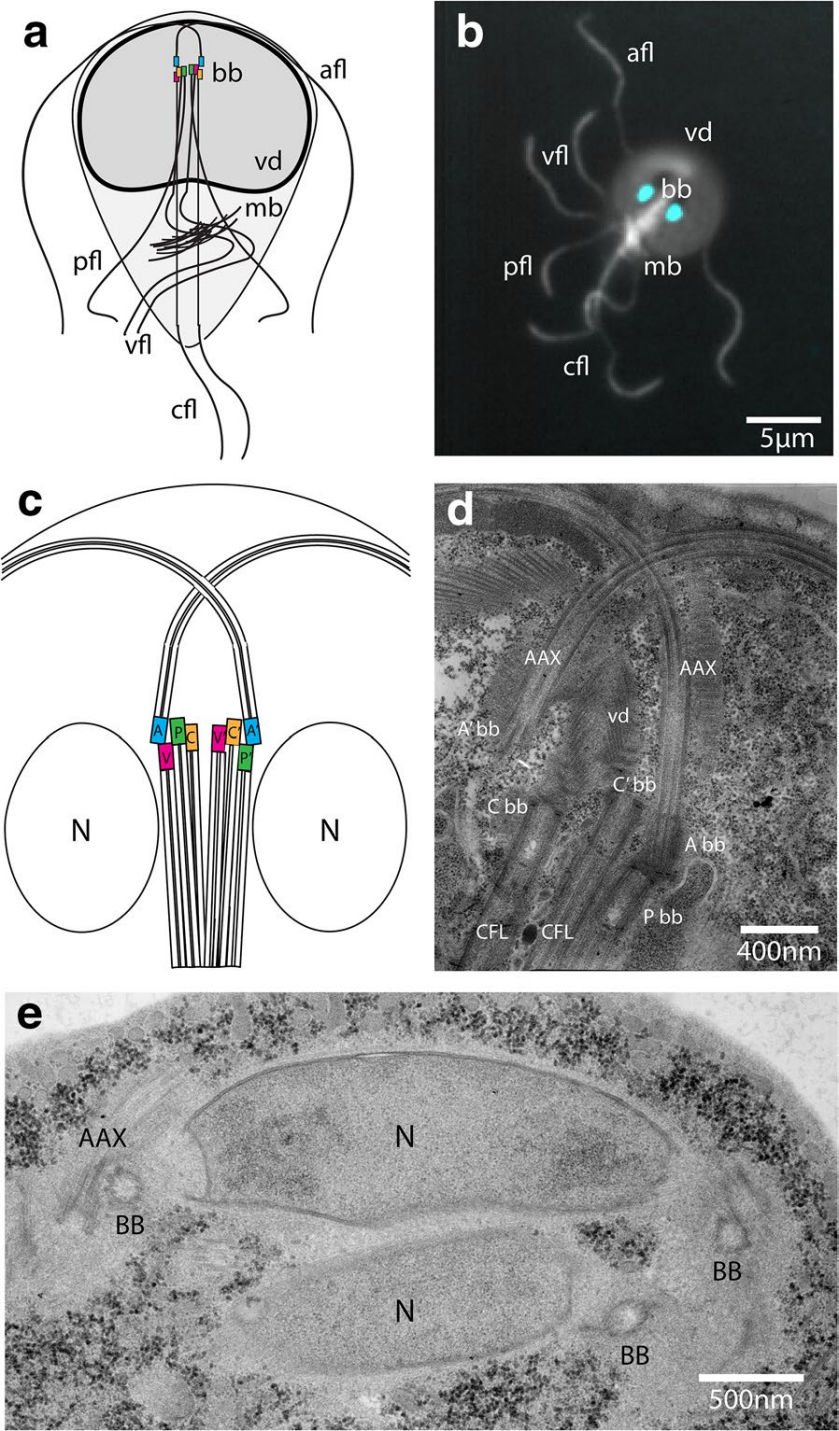
*Giardia* microtubule cytoskeleton emphasizing interphase basal body positions and migration during mitosis. A schematic representation of the characteristic teardrop shape of *Giardia* and the cytoskeletal features of the cell is shown in panel (**a**), including: the basal bodies (bb), four pairs of flagella (afl = anterior, cfl = caudal, pfl = posteriolateral, vfl = ventral), median body (mb), and ventral disc (vd). Anti-tubulin immunostaining reveals the cytoplasmic lengths of all eight flagella, which begin at the basal bodies located between the two nuclei labeled with DAPI (**b**). Panel **c** shows a schematic of the basal body tetrads arrangements and their association with specific flagellar axonemes (A/A’ = anterior, C/C’ = caudal, P/P’ = posteriolateral, V/V’ = ventral, N = nuclei). A transmission electron micrograph (TEM) of the anterior region of the cell in panel **d** shows the organization the basal bodies and their associated flagellar axonemes (Abb/A’bb = anterior basal body, Cbb/C’bb = caudal basal body, Pbb = posteriolateral basal body, and AAX = anterior axonemes). The ventral disc is also nucleated from the caudal basal bodies. A TEM cross section of a mitotic cell in panel **e** shows the migration of basal bodies from their interphase position between the two nuclei (panel **c**) to the spindle poles, where they are associated with the spindle microtubules and flagellar axonemes (i.e., one anterior axoneme (AAX) is visible)

The swimming form of *Giardia*, or the “trophozoite,” has eight flagella that retain the canonical “9 + 2” structure of a motile flagellum [12]. Each flagellum also has radial spokes, dynein arms, and outer doublet and central pair microtubules [13, 14]. The eight flagella are organized into four bilaterally symmetrical pairs: the anterior, the caudal, the posteriolateral, and the ventral (Fig. 1). The basal bodies for all flagella are located in the anterior of the cell between the two nuclei. Each flagellar pair differs in its cytological position within the trophozoite and in its association with ancillary structures. The coordinated beating of *Giardia’s* eight motile flagella results in complex movements essential for motility and cell division, and may aid in parasite attachment to the host gut epithelium [15, 16]; however, not all flagellar pairs have characteristic flagellar waveforms [15].

In general, eukaryotic flagella extend from a basal body or centriole and are surrounded by a specialized flagellar membrane after they project from the cell surface. In contrast to other flagellated protists, each of the eight *Giardia* axonemes has a long cytoplasmic region that extends from a centrally located basal body before exiting the cell body as a membrane-bound flagellum (Fig. 1 and see [16]). The ratio of the length of the cytoplasmic region to the membrane-bound portion varies between each flagellar pair (e.g., over two-thirds of the length of the caudal axonemes is in the cytoplasmic region, whereas only a third of the anterior axoneme is cytoplasmic). The anterior axonemes cross over the ventral disc spiral before exiting on the right and left sides of the anterior region of the cell. The distance from the exit point from the cell body to the flagellar tip is about 12 µm. Running longitudinally along the anterior-posterior axis of the cell, the two caudal axonemes exit the cell body and extend about 7 µm at the posterior end. The ventral axonemes exit and extend about 14 µm on the ventral side in the “lateral shield” region posterior to the disc. The posteriolateral axonemes angle outward at the lower third of the cell body, extending about 8 µm from the cell body. Electron-dense “ciliary pockets” are found at the regions where each flagellum exits the cell body [17]. This review presents detailed findings concerning the structure, duplication, and migration of the eight unique *Giardia* basal bodies during the parasite life cycle.

## Structure and positions of the eight basal bodies

The eight flagellar basal bodies that nucleate the axonemes are positioned between the two nuclei in the cell interior (Fig. 1). The long cytoplasmic regions of the axonemes are not extended transition zones; basal body transition zones are restricted to small regions proximal to the basal bodies rather than to the entire cytoplasmic axoneme [17]. The anterior basal bodies are located toward the anterior ends of the two nuclei and oriented toward the anterior end of the cell. Basal bodies that nucleate the ventral, caudal, and posteriolateral axonemes are positioned posteriorly below the two anterior basal bodies and are oriented toward the posterior of the cell. Interphase trophozoites lack both barren and probasal bodies [18].

Flagellar and basal body proteomics in *Giardia* have contributed to our overall understanding of flagellar structure and evolution; however, the selective isolation of axonemes or basal bodies from the extensive cytoskeleton in *Giardia* has proved to be challenging [19]. Canonical basal body-associated proteins (e.g., centrin, delta-tubulin and epsilon tubulin) and some components of the BBSome are present in the *Giardia* genome (Table 1). Centrin localizes to two distinct clusters adjacent to the two nuclei during interphase, colocalizing with the flagellar basal bodies [20]. Consistent with observations in other flagellated cells, gamma-tubulin also localizes to flagellar basal bodies during interphase; however, gamma-tubulin localization is restricted only to flagella that are newly produced during cell division [18].

**Table 1:** Known and candidate *Giardia* basal body proteins including supporting evidence

GiardiaDB	Protein name	Protein family	PFAM	Basal body and/or other subcellular localization	Evidence
GL50803_6744	Centrin	Centrin	PF13499; PF13833	All basal bodies	HOM, IFA
GL50803_104685	Caltractin	Caltractin	PF13499	All basal bodies	HOM, IFA, GFP
GL50803_114218	Gamma-tubulin	Tubulin	PF00091; PF03953	All basal bodies, cytoplasm	HOM, PRO, IFA, GFP
GL50803_5462	Delta-tubulin	Tubulin	PF00091	Cytoplasm	HOM, GFP
GL50803_6336	Epsilon-tubulin	Tubulin	PF00091	Cytoplasm	HOM, GFP
GL50803_17429	GCP2	Spc97 Spc98	PF04130	n.d.	HOM
GL50803_12057	GCP3	Spc97_Spc98	PF04130	All basal bodies, median body, cytoplasmic caudal, posteriolateral, and anterior axonemes	HOM, GFP
GL50803_104150	Kinase, PLK	Ser/Thr kinase domain, POLO box domain	PF00069, PF00659	All basal bodies	EPI
GL50803_4689	Hypothetical protein	None	None	All basal bodies	PRO, EPI
GL50803_4692	Hypothetical protein	None	None	All basal bodies	PRO, EPI
GL50803_41512	Flagella associated protein Rib72	DUF1126 domain of unknown function	PF06565	All basal bodies, cytoplasm	PRO, EPI
GL50803_114546	Hypothetical protein	None	None	All basal bodies, all cytoplasmic axonemes	GFP
GL50803_3582	DUF390 domain containing protein	DUF390 domain of unknown function	PF04094	All basal bodies, all cytoplasmic axonemes	GFP
GL50803_8974	Hypothetical protein	None	None	All basal bodies, all cytoplasmic axonemes, median body, ciliary pocket	GFP
GL50803_16935	Hypothetical protein	None	None	All basal bodies, all flagella, ventral disc lateral crest, median body	GFP
GL50803_15218	WD-40 repeat protein	WD40 repeats	PF00400	All basal bodies, all flagella, ventral disc lateral crest, median body	PRO, IFA, GFP
GL50803_9117	CAMP-dependent protein kinase regulatory chain	Cyclic nucleotide binding domain	PF00027	All basal bodies, anterior axonemes, caudal axonemes	EPI
GL50803_11214	Kinase, AGC PKA	Kinase, AGC PKA	PF00069	All basal bodies, anterior axonemes, caudal axonemes	EPI
GL50803_16202	Axoneme central apparatus protein PF16/SPAG6	Central pair associated protein	PF00514	All basal bodies, anterior axonemes, posteriorlateral axonemes, caudal axonemes, median body	PRO, IFA, EPI
GL50803_11867	RIB43A	RIB43A	PF05914	All basal bodies, anterior axonemes, posteriorlateral axonemes, median body	EPI
GL50803_7351	Hypothetical protein	None	None	All basal bodies, ciliary pocket, cytoplasmic ventral, caudal, posteriolateral, and anterior axonemes	GFP
GL50803_24412	Ankyrin-repeat domain containing protein	Ankyrin-repeat domains	PF12796	All basal bodies, cytoplasmic anterior and posteriolateral axonemes	GFP
GL50803_10460	Hypothetical protein	None	None	All basal bodies, cytoplasmic anterior axonemes, median body	GFP
GL50803_17586	Ankyrin-repeat domain containing protein	Ankyrin-repeat domains	PF12796	All basal bodies, cytoplasmic anterior, posteriolateral and ventral axonemes	GFP
GL50803_7192	FWWh domain containing protein	FWWh domain of unknown function	PF14922	All basal bodies, cytoplasmic caudal axonemes, posteriolateral and anterior axonemes	GFP
GL50803_8557	Hypothetical protein	None	None	All basal bodies, cytoplasmic caudal axonemes, posteriolateral and anterior axonemes	GFP
GL50803_102455	GiKIN6a	Kinesin-6	PF00225	All basal bodies, cytoplasmic posteriolateral and anterior axonemes, median body, ventral disc	GFP
GL50803_11775	Kinase, ankyrin-repeat domain containing protein	Ser/Thr kinase domain, ankyrin-repeat domains	PF00069, PF1279	All basal bodies, cytoplasmic posteriolateral and caudal axonemes	GFP
GL50803_15219	Macoilin domain containing protein	Macoilin	PF09726	All basal bodies, cytoplasmic posteriolateral axonemes, cytoplasm	GFP
GL50803_15446	Hypothetical protein	None	None	All basal bodies, cytoplasmic posteriolateral axonemes, median body	GFP
GL50803_6709	Hypothetical protein	None	None	All basal bodies, median body, all flagella, ventral disc lateral crest	GFP
GL50803_92498	Kinase, NEK	Kinase, NEK	PF00069	All basal bodies, median body, anterior axonemes, posteriorlateral axonemes, caudal axonemes, ventral disc	EPI
GL50803_17154	RRP7 domain containing protein	Ribosomal RNA-processing protein 7 (RRP7) domain	PF12923	All basal bodies, median body, cytoplasmic anterior axonemes, caudal axonemes, posteriolateral axonemes	GFP
GL50803_4624	DUF4490 domain containing protein	DUF4490	PF14892	All basal bodies, median body, cytoplasmic caudal, ventral, posteriolateral and anterior axonemes	GFP
GL50803_13352	T-complex protein-10/Sas-4/CENPJ	T complex protein 10 family C terminal domain	PF07202	All basal bodies, median body, cytoplasmic posteriolateral axonemes, ciliary pocket	PRO, IFA, GFP
GL50803_10232	LRR repeat domain containing protein	CENP-F_leu_zip leucine-rich repeats	PF10473	All basal bodies, median body, ventral disc	GFP
GL50803_31671	RPB7 SHS2 domain containing protein	N terminal SHS2 domain of RNA pol II subunit RPB7	PF03876	All basal bodies, nuclei, cytoplasmic posteriolateral and anterior axonemes	GFP
GL50803_16220	Ankyrin-repeat domain containing protein	Ankyrin-repeat domains	PF12796	All basal bodies, plasma membrane, cytoplasm	GFP
GL50803_16973	Hypothetical protein	None	None	All basal bodies, posteriorlateral axonemes, median body	PRO, IFA
GL50803_17096	Ankyrin-repeat domain containing protein	Ankyrin-repeat domains	PF12796	All basal bodies, ventral disc lateral crest	GFP
GL50803_16424	MiflIP domain containing protein	Mlf1IP	PF10248	All basal bodies, ventral disc lateral crest, cytoplasmic anterior axonemes	GFP
GL50803_17097	Ankyrin-repeat domain containing protein	Ankyrin-repeat domains	PF12796	All basal bodies, ventral disc lateral crest, cytoplasmic anterior axonemes, median body	GFP
GL50803_101326	Hypothetical protein	None	None	All basal bodies, ventral disc lateral crest, cytoplasmic caudal axonemes	GFP
GL50803_15499	DUF390 domain containing protein	DUF390 domain of unknown function	PF04094	All basal bodies, ventral disc lateral crest, cytoplasmic posteriolateral axonemes	GFP
GL50803_5568	DUF866 domain containing protein	DUF866	PF05907	All basal bodies, ventral disc lateral crest, ventral axonemes	GFP
GL50803_5010	Ser/Thr phosphatase PP2A-2 catalytic subunit	Metallo-dependent phosphatases	PF00149	All basal bodies, ventral disc, anterior axonemes, posteriorlateral axonemes, caudal axonemes	PRO, IFA
GL50803_17563	Kinase, CMGC MAPK	Kinase, CMGC MAPK	PF00069	All basal bodies, ventral disc, caudal axonemes, median body	EPI
GL50803_16279	Kinase, NEK	Kinase, NEK	PF00069	All basal bodies, ventral disc, caudal axonemes, posteriorlateral axonemes	EPI
GL50803_5358	Aurora kinase	Aurora kinase	PF00069	All basal bodies, ventral disc, nuclei, anterior axonemes, median body	EPI
GL50803_15193	Hypothetical protein	None	None	Some basal bodies	PRO, IFA, GFP
GL50803_29796	Hypothetical protein	None	None	Some basal bodies, anterior flagella, ventral flagella, median body	GFP
GL50803_6254	Hypothetical protein	None	None	Some basal bodies, cytoplasmic posteriolateral axonomes	GFP
GL50803_15455	PACRG1	Parkin coregulated protein	PF10274	Cytoplasm, nuclei	HOM, GFP
GL50803_14048	EB1	EBl-like C terminal motif	PF03271	Nuclear membrane, median body	GFP
GL50803_15956	FAP52	WD40 repeats	PF00400	Plasma membrane, all axonemes	HOM, GFP
GL50803_5333	Calmodulin	EF-hand domain	PF13499	n.d.	HOM
GL50803_33762	POC1	WD repeat protein	PF00400	n.d.	HOM
GL50803_32375	POC18	None	None	n.d.	HOM
GL50803_13372	FAP45	TPH domain	PF13868	n.d.	HOM
GL50803_5167	VFL3	None	None	n.d.	HOM
GL50803_15248	Bub2	TBC domain	PF00566	n.d.	HOM
GL50803_8738	BBS1	BBS1	PF14779	n.d.	HOM
GL50803_23934	BBS2	None	None	n.d.	HOM
GL50803_10529	BBS4	TPR_1 Tetratricopeptide repeat	PF01515	n.d.	HOM
GL50803_8146	BBS5	DUF1448 domain of unknown function	PF07289	n.d.	HOM
GL50803_8508	BBS8	TPR_1 Tetratricopeptide repeat	PF01515	n.d.	HOM

Evidence for candidate basal body proteins is based on homology to known basal body proteins in other organisms (HOM) [9, 41, 42], enrichments of *Giardia* basal body proteins in proteomic analyses (PRO) [19, 41] and/or subcellular localization using either heterologous antibodies (IFA) [25–27, 43, 44] or epitope-tagging (EPI) [19, 41]. Candidate basal body proteins identified with an ongoing, publically available, *Giardia* C-terminal GFP-tagging project for EuPATHDB [45] in our laboratory are also presented (GFP)

**Fig. 2:**
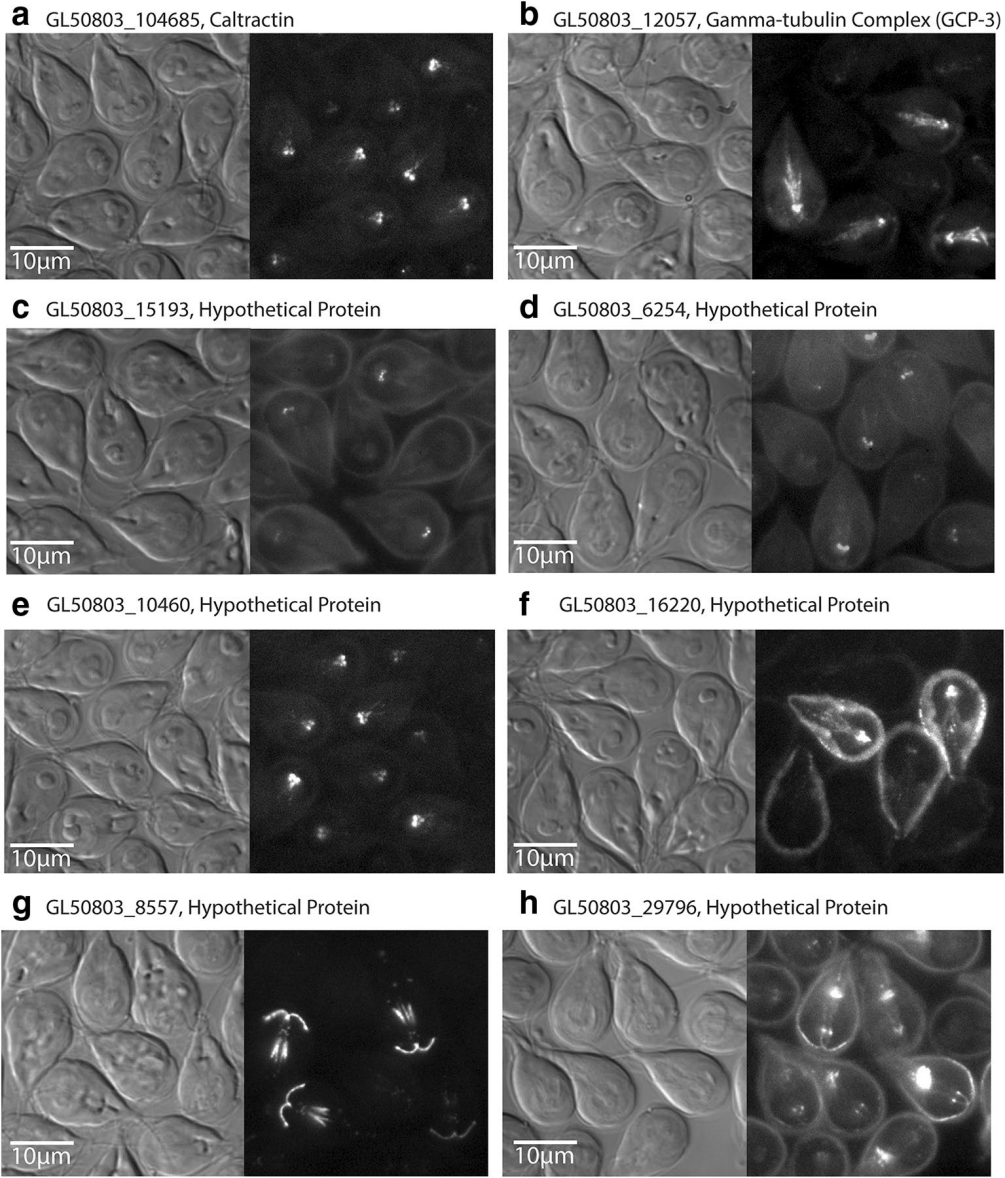
Representative GFP-tagged basal body proteins in *Giardia.* Many known basal body proteins, including caltractin (**a**) and gamma-tubulin complex 3 (**b**), have been identified in the *Giardia* genome by homology and confirmed by GFP tagging to localize to the basal bodies. *Giardia* also has proteins that localize to some basal bodies (**c**, **d**) or all basal bodies (**e**), but lack homology to known basal body proteins. Further, proteins that localize to basal bodies and other structures, including the cell membrane (**f**), the cytoplasmic axonemes (**g**), and the median body (**h**), have also been identified

 Notably, more than 1000 hypothetical proteins (e.g., those lacking significant similarity to proteins in other organisms) have been identified from the *Giardia* genome, and this genetic novelty is reflected in the analyses of basal body [19] and cytoskeletal proteomes [21]. Proteins localizing to basal bodies may be structural components or may merely dock at basal bodies before they are transported to other parts of the cell. Many known basal body proteins are confirmed in the *Giardia* genome by homology or from localization studies (see centrin (GL50803_6744) and caltractin (GL50803_104685) in Table 1 and imaged in Fig. 2). Other proteins identified as basal body proteins by comparative proteomics lack basal body localization in *Giardia* (e.g., FAP52 (GL50803_15956) and PACRG1 (GL50803_15455), see Table 1), or localize to other cytoskeletal structures in addition to the basal bodies (e.g., GL50803_8557 and GL50803_29796, see Table 1, and imaged in Fig. 2). Furthermore, *Giardia* has proteins that localize to some or all basal bodies, but lack homology to known basal body proteins (e.g., GL50803_15193 and GL50803_6254, see Table 1 and imaged in Fig. 2). In total, there are 49 proteins that localize to some or all *Giardia* basal bodies. Five components of the BBSome are present in *Giardia*, although localization of these to basal bodies has not been confirmed (Table 1).

## Additional basal body structures or accessories

Additional basal body structures or accessories have not been identified in *Giardia.* In trophozoites, however, complex axoneme-associated structures are associated with each flagellar pair ([22] and Fig. 1). These extra-axonemal structures confer a unique structural identity to each flagellar pair; thus, each pair has a unique functional role in parasite motility [15]. *Giardia* axoneme-associated structures include the “marginal plate” that is associated with the anterior axonemes [22]; the fin structures on the ventral axonemes [23]; the electron dense material on the posteriolateral axonemes, and the “caudal complex” or “funis” microtubules that surround the caudal axonemes. Undiscovered basal body structures may further distinguish and define each axoneme.

The ventral disc microtubules nucleate from the caudal basal bodies and extend to form the right-handed spiral array that mediates attachment to the host intestine during infection. Over 50 proteins associate with the ventral disc (e.g., disc-associated proteins or “DAPS”) as seen in proteomic and localization analyses [21].

## Origins of the eight basal bodies

The eight axonemal basal bodies are inherited by each daughter cell during a mitotic division in trophozoites that includes two spindles and four spindle poles [20]. In some flagellates, such as *Chlamydomonas*, flagella are resorbed at the onset of mitosis and the basal bodies (as centrioles) are recruited to function as part of the mitotic spindle poles [24]. Unlike *Chlamydomonas*, both centrin localization [25, 26] and ultrastructural studies [20] indicate that all eight flagella are retained during mitosis, and the flagella and their associated basal bodies migrate to the four spindle poles. Two of the eight flagellar basal bodies associate with each of the four spindle poles during the division of the two nuclei [20]. One basal body at each spindle pole acts as the central structural component of the MTOC, while a second basal body is observed at the periphery of the spindle pole region with an associated axoneme [20]. This peripheral basal body may play an indirect role in spindle nucleation, and its association with the spindle pole may ensure proper segregation to the daughter cells.

During the onset of prophase and spindle assembly, the number of centrin foci increases from two to four due the duplication or separation of the basal body tetrads [18, 20]. Spindle microtubule assembly begins with the appearance of microtubules near the duplicated basal bodies; these microtubules extend around each nucleus and continue to elongate as the nuclei migrate to the cell midline. Centrin foci are found at the sites of spindle nucleation during nuclear migration and move to the periphery of the nuclei as the spindle microtubules elongate. Importantly, centrin localizes to only the four basal bodies associated with the spindle poles [18]. Spindle microtubule elongation ceases by the end of prophase, when each spindle surrounds each nucleus and kinetochore microtubules of the spindle capture chromosomes through polar openings [20]. Upon completion of nuclear migration in prophase [27], the microtubules surrounding each nucleus form two independent bipolar spindles that are stacked in the dorsal–ventral plane in metaphase. At this time, centrin is localized to the four spindle poles, where it will remain throughout anaphase A and anaphase B. Gamma-tubulin staining returns during anaphase, but is limited to four of the eight basal bodies and was not observed at the spindle poles [27]. During telophase, the centrin foci at each spindle pole move from their anaphase position near the cell periphery to their interphase position between each pair of nuclei.

Migration of basal bodies and nuclei may be coordinated events facilitated by centrin-dependent attachment of basal bodies to the nuclear envelope. Migration of the nuclei to the center of the cell during prophase displaces the flagellar basal bodies causing a dramatic rearrangement of the flagella ([18] and Fig. 1). Most notably, the anterior flagella move along the cell periphery from one side of the cell to the other, which is in accordance with the migration of the associate basal body to the spindle poles [18]. Gamma-tubulin immunostaining of basal bodies is reported to disappear early in prophase and reappears during later mitotic stages. This behavior is unique among flagellated protists and resembles what is seen during the reassembly of functional centrosomes in animal cells. Furthermore, these observations suggest a possible association of gamma-tubulin and the kinetochore complex of *Giardia* [27].

## Basal body behavior during encystation and excystation

*Giardia* cysts are ingested from contaminated water sources and the parasite completes its life cycle in the small intestine of the host. After ingestion, the cyst transforms into a flagellated trophozoite that attaches to the intestinal villi and subsequently colonizes the small intestine. Attachment allows trophozoites to resist peristaltic flow in the gut [16] and is mediated by an elaborate microtubule structure termed the ventral disc [28, 29]. Flagellated trophozoites later develop into infectious cysts that are excreted and persist in the environment, disseminating the infection to other hosts [28, 30].

The cyst stage contains internalized flagella and is characterized by a thick cyst wall that enables resistance to environmental stresses [31]. During encystation the two nuclei divide without cytokinesis to form tetra-nucleated cysts with 8N ploidy [32]. A subsequent round of DNA replication increases the ploidy of mature cysts to 16N. Throughout the process of encystation, the arrangement of the flagellar apparatus in the majority of cysts is the same as what is observed in the interphase cell. Only a single flagellar apparatus, comprised of four flagellar pairs with associated basal bodies, is present and there is no duplication of either the flagellar apparatus or basal bodies. Ultrastructural analysis of mature cysts shows that basal body tetrads are arranged and localized between the pairs of daughter nuclei, with one nucleus from each pair associated with a basal body tetrad [31].

After a suitable host ingests the *Giardia* cyst, the parasite undergoes excystation in the small intestine to differentiate into the flagellated “excyzoite.” During excystation, the six flagella directed toward the posterior of the cell, namely the caudal, posteriolateral, and ventral flagella, protrude through the cyst wall allowing the cell to squeeze through this opening. The newly emerged excyzoite contains a single flagellar apparatus with basal bodies positioned similarly to the interphase trophozoite. While few molecular details are known about excystation, it is thought that the excyzoite undergoes two rapid, consecutive cell divisions to produce four trophozoites [31]. In the first division, the basal body tetrads segregate and localize between the pairs of nuclei, and the excyzoite undergoes cytokinesis but not nuclear division. The four intact nuclei resulting from this division are then segregated so that each daughter cell receives two nuclei with previously replicated DNA. Each daughter cell enters into mitotic division without DNA replication to form two trophozoites with two nuclei and two basal body tetrads that nucleate the eight flagella.

## Notable findings

*Giardia*’s eight basal bodies have a unique inheritance pattern in daughter cells. In the interphase trophozoite, eight basal bodies are arranged into two tetrads and each basal body pair is associated with a distinct flagellar pair. When the trophozoite is viewed dorsally, the left tetrad consists of anterior/ventral and caudal/posteriolateral basal bodies, while the right tetrad consists of caudal/ventral and anterior/posteriolateral basal bodies (Fig. 1). The polarity of each daughter cell is thought to be determined through the association of axonemal basal bodies with the dividing nuclei [20]. During division, eight parent flagella persist and are inherited in a semi-conservative manner, with each progeny receiving four flagella from the parent cell while four complementary flagella are assembled de novo in each cell. Importantly, these de novo flagella lack polyglycylated tubulin, which makes this post-translational modification a convenient marker of the parent (inherited) flagella. Thus, the organization of basal body pairs in *Giardia* informs our understanding of the mitotic distribution of the eight flagella to two daughter cells.

Due to the inheritance and de novo assembly of specific flagella in daughter cells, a multigenerational division cycle has been proposed wherein the relative age of a flagellar axoneme is different based on its anatomical position in the trophozoite [18]. The flagella of some other protists are known to undergo a similar maturation process that takes more than one cell cycle [33], mirroring the behavior of centrioles in metazoans (reviewed in [34]). Based on immunostaining with a polyglycylated tubulin antibody to visualize parental axonemes and an acetylated tubulin antibody to visualize daughter axonemes, eight parental (old) flagella are retained and eight new flagella are synthesized each cell division cycle [18]. Before mitosis is completed, flagellar and basal body duplication occurs [18, 20]. Flagellar regeneration begins in anaphase with short flagella (presumably the new ventral and posteriolateral pairs) emerging from the spindle poles [18, 20]. While specific molecular markers have not been used to track each flagellar pair to confirm their identity during division [18], the full length parental anterior axonemes are proposed to become the right caudal axonemes in the new daughter cells. Parental right caudal axonemes are then proposed to become the left caudal axonemes. Thus each daughter cell inherits a full complement of eight axonemes and associated basal bodies—four parental (old), and four newly duplicated each generation [18, 20].

The division of the caudal axonemes and their associated basal bodies also has notable implications for the de novo nucleation and assembly of the daughter ventral discs. After the daughter nuclei are partitioned and the caudal flagellar basal bodies have been repositioned between the two nuclei [18], two new dorsal daughter ventral discs are assembled during telophase. The parental ventral disc is not disassembled until later in the cell cycle. Thus, the caudal basal bodies nucleate the caudal axonemes and also determine the site of ventral disc assembly, establishing the polarity of the new daughter cells. The left caudal flagellum alone has been proposed to nucleate the spiral MT arrays that form the basis of the ventral disc [22]; however, recent work shows that both caudal basal bodies nucleate the ventral disc MTs (see Fig. 1). Live imaging is required to confirm flagellar migration during cell division and ultimately characterize the forces and mechanisms involved in flagellar maturation and daughter disc nucleation. The timing and mechanism by which the extra-axonemal-associated structures (e.g., marginal plate, caudal complex or funis) are assembled during cell division also remains unclear [17].

## Conclusions

While some cellular functions of the cytoskeleton are conserved across eukaryotes (e.g., mitosis and cytokinesis), the molecular components and pathways underlying these processes have extensive variation in less well-studied eukaryotic groups [35]. Based on their evolutionary distances and the complex composition of their diverse cytoskeletal structures, excavate protists such as *Giardia* may represent an undiscovered reservoir of novel basal body-associated proteins.

The composition, fundamental structure, and functional properties of the eight *Giardia* basal bodies remain to be explored. We currently lack high-resolution images that distinguish the differences between each individual basal body. We also lack molecular characterizations of any basal body-associated structures during interphase, flagellar duplication, or mitotic division. *Giardia* basal bodies have unique identities and spatial positions in the cell, and are likely of differing ages due to the mode of basal body inheritance. We have no structural detail that might provide clues linking position or age to particular basal bodies. In addition to trophozoite mitosis, both encystation and excystation are two life cycle transitions that could illuminate basal body duplication, migration, and partitioning into daughter cells. Initial cytological and ultrastructural analyses of the basal bodies and flagellar structures should be updated and revisited at higher resolution using current state of the art fixation techniques and imaging methods, as have been recently used in the analysis of the ventral disc.

Due to our current inability to genetically manipulate *Giardia*, studying giardial protein function is challenging [29, 36]. A complete genome and reverse genetic tools to generate dominant negative mutants [37, 38] or antisense [39] and morpholino-based knockdowns [40] have permitted the identification and characterization of novel structural components and the mechanisms underlying *Giardia*’s cytoskeletal dynamics [40]. Current or future genetic methods could be applied to the study of basal body proteins, and the availability of a more complete inventory of basal body-localizing proteins in *Giardia* will facilitate these efforts.

## Abbreviation

MTOC: microtubule organizing center
